# Supplementary figures and images for: Realgar Alleviated Neuroinflammation Induced by High Protein and High Calorie Diet in Rats via the Microbiota-Gut-Brain Axis
Source: Nutrients. 2022 Sep 23;14(19):3958. doi: 10.3390/nu14193958 (PMC9572528; doi:10.3390/nu14193958)

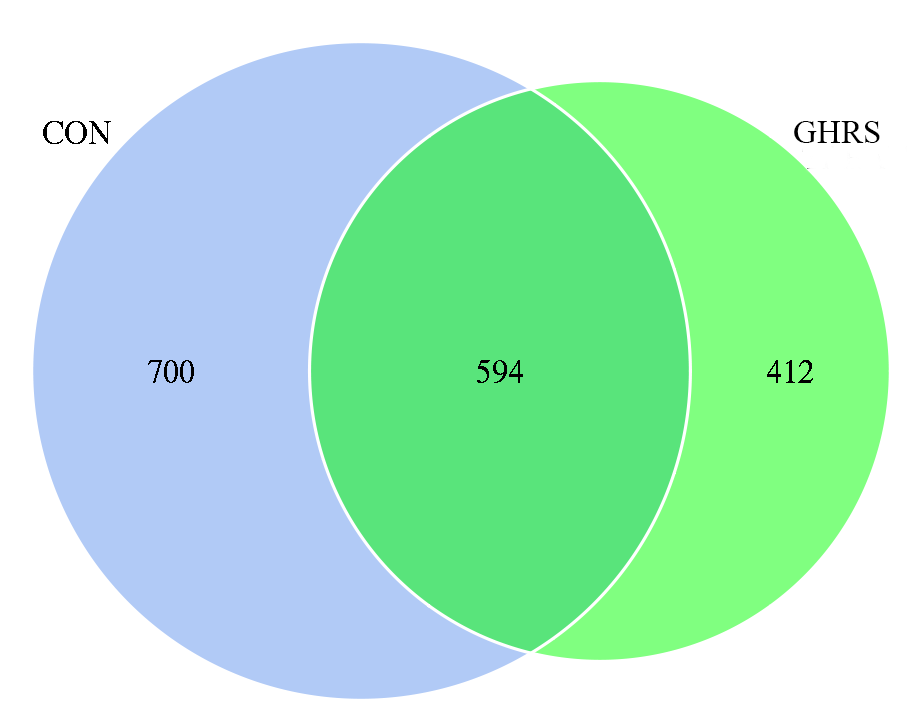

Supplement: Supplementary file 1 [file nutrients-14-03958-s001.zip › Suppl 1.tif]

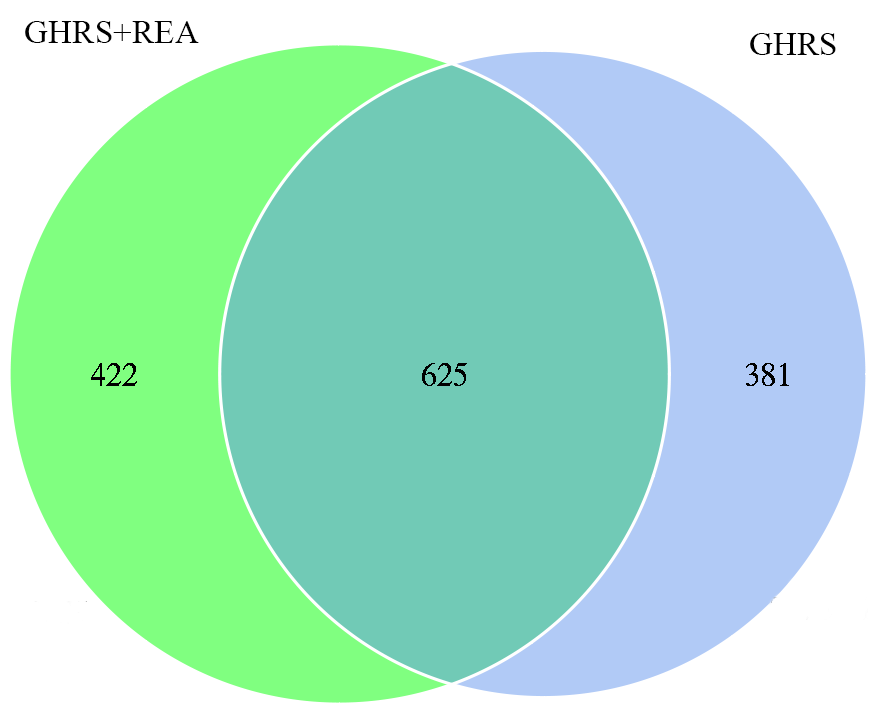

Supplement: Supplementary file 1 [file nutrients-14-03958-s001.zip › Suppl 2.tif]
